# CTNNB1 S45F Mutation Predicts Poor Efficacy of Meloxicam Treatment for Desmoid Tumors: A Pilot Study

**DOI:** 10.1371/journal.pone.0096391

**Published:** 2014-05-01

**Authors:** Shunsuke Hamada, Naohisa Futamura, Kunihiro Ikuta, Hiroshi Urakawa, Eiji Kozawa, Naoki Ishiguro, Yoshihiro Nishida

**Affiliations:** Department of Orthopaedic Surgery, Nagoya University Graduate School and School of Medicine, Nagoya, Japan; Johns Hopkins University, United States of America

## Abstract

We hypothesized that patterns of CTNNB1 (β-catenin) mutations would affect the outcome of conservative therapy in patients with desmoid tumors. This study aimed to determine the significance of CTNNB1 (β-catenin) mutations in predicting the treatment outcome in patients with desmoid tumors treated with meloxicam, a cyclooxygenase-2 (COX-2) selective inhibitor. Between 2003 and 2012, consecutive thirty-three patients with extra-peritoneal sporadic desmoid tumors were prospectively treated with meloxicam as the initial systemic medical therapy. The efficacy of meloxicam was evaluated according to Response Evaluation Criteria in Solid Tumors (RECIST). DNA was isolated from frozen tissue or formalin-fixed materials. CTNNB1 mutation analysis was performed by direct sequencing. Positivity of nuclear β-catenin staining by immunohistochemistry was compared with the status of CTNNB1 mutations. The correlation between the efficacy of meloxicam treatment and status of CTNNB1 mutations was analyzed. Of the 33 patients with meloxicam treatment, one showed complete remission (CR), 7 partial remission (PR), 12 stable disease (SD), and 13 progressive disease (PD). The following 3 point mutations were identified in 21 of the 33 cases (64%): T41A (16 cases), S45F (4 cases) and S45P (one case). The nuclear expression of β-catenin correlated significantly with CTNNB1 mutation status (p = 0.035); all four cases with S45F mutation exhibited strong nuclear expression of β-catenin. S45F mutation was significantly associated with a poor response (all cases; PD) (p = 0.017), whereas the other mutations had no impact on efficacy. The CTNNB1 mutation status was of significant prognostic value for meloxicam treatment in patients with sporadic desmoid tumors.

## Introduction

Desmoid tumors, also known as aggressive fibromatosis, are mesenchymal tumors that show marked local aggressiveness, but rarely metastasize, and do not cause disease-specific death if they are not located at anatomically critical sites [1], [2]. Extra-peritoneal desmoid tumors, which are usually sporadic in nature, occur across a wide age range, and can arise at virtually any body site. Extensive surgical resection has been the standard treatment for decades. However, radical surgical intervention and/or repeated surgery due to a high recurrence rate (range 34–53% at 5 years) [3], [4] occasionally lead to significant treatment-related morbidity including amputation or significant functional impairment. Several reports have failed to demonstrate the significance of margin status in surgery for local recurrence [3]–[5], while spontaneous regression has also been reported [5], [6]. Several authors recently demonstrated the effectiveness of conservative treatment for desmoid tumors including radiotherapy and pharmacological treatment [7], [8]. Pharmacological treatment includes anti-hormone, NSAIDs, and targeted and traditional cytotoxic chemotherapies [2], [9]–[12]. However, the efficacy of these treatments cannot be predicted, and so remains a crucial problem.

β-catenin plays various important roles in the tumorigenesis of desmoid tumors, and has a diagnostic potential to differentiate them from other lesions [13], [14]. The nuclear accumulation of β-catenin causes activation of Wnt signaling, and in turn transcription of target genes in fibroproliferative disease [15]. Most desmoid tumors arise sporadically, with a minority associated with familial adenomatous polyposis (FAP), which is caused by a germline mutation of the adenomatous polyposis (APC) gene [15]. APC protein forms β-catenin destruction complex and is involved in the regulation of Wnt signaling. Several recent studies have reported point mutations of CTNNB1 (β-catenin) exon3. These mutations, occurring at codon 41 and 45, were found in about 64–85% of all sporadic desmoids, with p.T41A (threonine to alanine), p.S45F (serine to phenylalanine), and p.S45P (serine to proline) being the most frequent ones [16]–[19]. These mutations were considered to lead to stabilization of β-catenin and tumorigenesis in desmoid tumors, suggesting that the status of CTNNB1 mutations might influence the efficacy of various treatments for patients with these tumors.

We previously reported the clinical results of consecutive patients prospectively treated with meloxicam, a cyclooxygenase-2 selective inhibitor [12], [20], [21]. The efficacy of meloxicam treatment varied among patients, indicating that biological markers are required to predict its efficacy in individual patients. Because consecutive patients were prospectively treated with the same medication, meloxicam, without other treatments, this cohort is particularly suitable to identify a biological marker predictive of the efficacy of this treatment. The aims of this study were to prospectively analyze the status of CTNNB1 mutations in consecutive patients with extra-peritoneal desmoid tumors treated with meloxicam, and to determine the significance of mutational status in predicting the efficacy of meloxicam treatment.

## Materials and Methods

### Ethics statement

The study protocol was approved by the Institutional Review Board (IRB) of Center for Advanced Medicine and Clinical Research, Nagoya University (approval ID: 1322), and written informed consent was obtained from all participants before the study commenced. The individuals in this manuscript have given written informed consent to publish these case details

### Patients and Tumor Tissues

There were 41 consecutive cases with extra-abdominal or abdominal wall desmoid tumors diagnosed in our institutions since 2003. They were all prospectively treated with meloxicam, a selective COX-2 inhibitor. Eight cases were excluded from this study. Three cases had been followed for less than 6 months, and two refused meloxicam treatment. In two cases, biopsy specimens were not available for analysis. One case was subsequently correctly diagnosed with another disease. There was no case with FAP-associated desmoid tumor. Finally, this study was composed of thirty-three consecutive patients with extra-peritoneal desmoid tumor prospectively treated with meloxicam without any other treatment previously. No patients received other medical treatments or radiotherapy during the meloxicam treatment. All thirty-three cases were histologically evaluated as having desmoid tumor by specialized pathologists.

### Efficacy of meloxicam treatment

As described in our previous study [12], meloxicam was administered orally at 10 mg/day. Baseline imaging of desmoid tumors by magnetic resonance imaging (MRI) and/or computed tomography (CT) was obtained before starting treatment. Patients treated with meloxicam were followed with physical and radiological examinations with MRI and/or CT at the outpatient unit of our institution every 3 to 6 months. The efficacy of meloxicam was evaluated according to Response Evaluation Criteria in Solid Tumors (RECIST) measured with MRI or CT at the latest follow-up or the end of meloxicam treatment. [22]. When patients were evaluated as showing a complete response (CR), they discontinued meloxicam. Patients with partial response (PR) or stable disease (SD) continued meloxicam. Patients with evaluation of PD could stop this treatment and chose other treatment modalities including low-dose chemotherapy or surgery depending on the tumor status, including location and aggressiveness. Considering that meloxicam has minimal side effects, SD status is thought to be preferable. Patients were divided into two groups, namely a favorable group (CR, PR, SD) and unfavorable group (PD). Age, gender, site, tumor size, follow-up period, and mutational status of CTNNB1 were examined as possible prognostic factors for responsiveness to meloxicam.

### Mutation analysis of CTNNB1

All patients received needle or incisional biopsy for histological diagnosis. Some of the obtained specimens were snap-frozen, and stored at −80°C for DNA and/or RNA analyses. DNA was extracted from frozen tissue or 5-µm-thick formalin-fixed, paraffin-embedded tissue by the High Pure PCR Template Preparation Kit (Roche Molecular Diagnostics, Mannheim, Germany), according to the manufacturer's instructions. DNA was first amplified by polymerase chain reaction (PCR) of 40 cycles at an annealing temperature of 58°C using LightCycler 480 System (Roche). To analyze the existence of point mutations in codons 41 or 45 of CTNNB1 exon 3, we designed 2 pairs of primers: forward 5′- GATTTGATGGAGTTGGACATGG-3′, reverse 5′-TCTTCCTCAGGATTGCCTT-3′, and forward 5′- TGGAACCAGACAGAAAAGCG-3′, reverse 5′- TCAGGATTGCCTTTACCACTC -3′. The expected sizes of the amplified products were 149 and 118 bp, respectively. PCR products were isolated by gel electrophoresis in 2% agarose, and amplified bands were extracted and purified using the QIAquick gel extraction kit (Qiagen, Valencia, CA). Purified products were subjected to direct sequencing using the above primers (forward), with Applied Biosystems Big Dye Terminator V3.1, and Applied Biosystems 3730x DNA analyzer (Applied Biosystems, Foster City, CA) at FASMAC Co. Ltd. (Kanagawa, Japan). All sequencing results were compared with those in the databases of NCBI-BLAST to confirm the mutation sites.

### Immunohistochemistry

Biopsy specimens fixed in 10% formalin and embedded in paraffin were subjected to immunohistochemical study for β-catenin. As described previously [23], the slides were incubated for overnight at 4°C with anti-human β-catenin mouse monoclonal antibody (M3539; Dako, Carpinteria, CA; dilution, 1∶200 dilution), and counterstained with hematoxylin, dehydrated, and mounted [24]. Nuclear positivity of β-catenin was evaluated by two independent observer (S. H., N. F.) without any knowledge of the clinicopathological information, and divided into 4 groups; 0% for positive stainable cell number (negative; 0), 1% to 9% (weak; 1+), 10% to 50% (moderate; 2+) and 51% to 100% (strong; 3+) on 10 independent high-power fields. The relationship between mutation status of β-catenin and nuclear positivity of β-catenin was analyzed.

### Statistical evaluation

Data were analyzed using the Fisher's exact test for dichotomous variables to examine correlations between the efficacy of meloxicam and clinical characteristics and between the mutation status of CTNNB1 and clinicopathological characteristics. Continuous variables of age and tumor size were compared between the two groups using the Mann-Whitney U test and between the multinomial groups using one-way analysis of variance. All statistical analyses were performed using SPSS version 20. P<0.05 was considered significant.

## Results

### Clinical features and efficacy of meloxicam treatment

Of the 33 patients prospectively treated with meloxicam, 11 were male, and 22 were female. The mean age was 41.6 years (median, 37.0 years; range, 10–74 years). The anatomic distribution of the tumors was the abdominal wall in 7 patients, neck 5, forearm and back 4 each, chest wall and thigh 3 each, shoulder 2, and upper arm, calf, foot, groin and retroperitoneum one each. The diameter of the tumor ranged from 20 to 220 mm (mean, 84 mm; median, 72 mm). No patients had received radiotherapy or other treatment for desmoid tumors prior to the meloxicam treatment. The median follow up was 36.6 months (10–120 months). The median period of medication was 15.2 months (range, 3 to 113 months). Of the 33 patients evaluated, there was one patient with CR, 7 with PR, 12 with SD, and 13 with PD. Of the 13 cases with PD, three were subjected to surgical treatment, and 8 to low-dose and/or doxorubicin-based chemotherapy. There were no significant differences in gender (p = 0.46), age (p = 0.34), tumor size (p = 0.63), or site of involvement (p = 0.22) between the favorable and unfavorable groups (Table 1).

**Table 1:** Patient characteristics between two prognosis groups.

Variables	Favorable group (n = 20)	Unfavorable group (n = 13)	P value
Gender			0.46
Female	12 (60%)	10 (77%)	
Male	8 (40%)	3 (23%)	
Mean age, years (range)	36.8 (10–74)	44.6 (20–73)	0.34
Mean size, mm (range)	84.5 (23–159)	83.0 (20–220)	0.63
Median follow up, months (range)	35.0 (10–120)	36.9 (13–85)	0.83
Site			0.22
Abdominal wall	3 (15%)	4 (31%)	
Other trunk	5 (25%)	4 (31%)	
Extremities	10 (50%)	2 (23%)	
Neck	2 (10%)	3 (15%)	

### Mutation status of CTNNB1 and efficacy of meloxicam treatment

**Figure 1:**
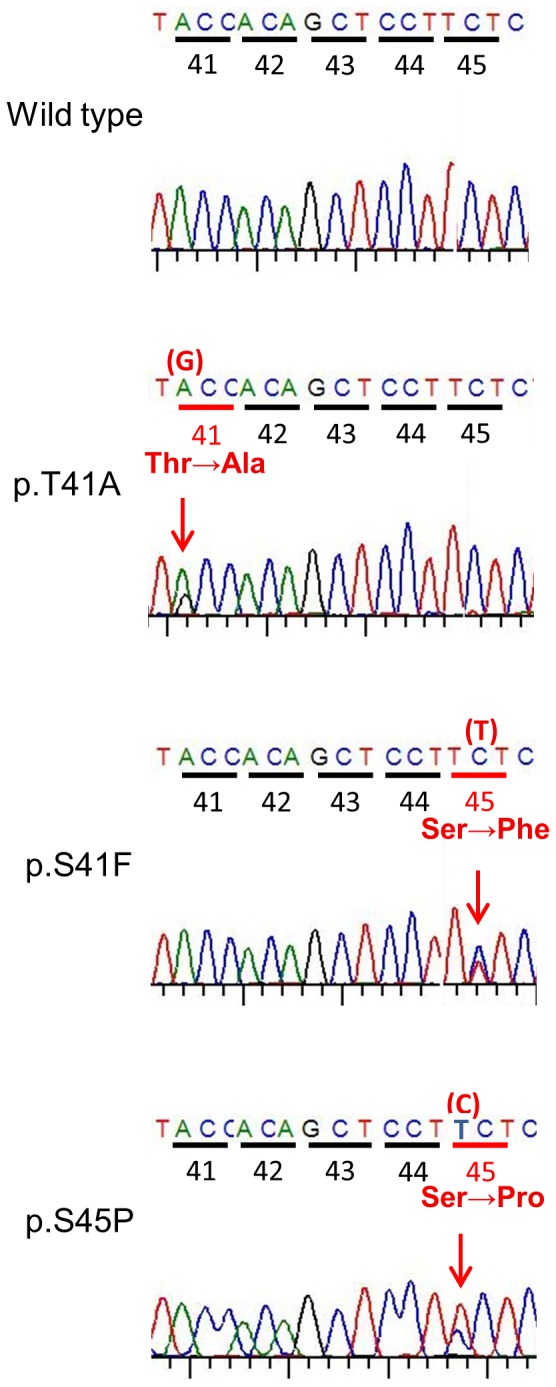
Representative sequencing results of each mutational type. Thr; Threonine, Ala; Alanine, Ser; Serine, Phe; Phenylalanine, Pro; Proline.

We performed genotyping of CTNNB1 exon 3 for all the cases. Point mutations were identified in 21 of the 33 cases (64%), and occurred in only 2 codons (41 and 45). Replacement of threonine by alanine (T41A) in codon 41 was detected in 16 cases (49%). Replacement of serine by phenylalanine (S45F) in codon 45 was detected in 4 (12.1%), and replacement of serine by proline (S45P) in codon 45 was detected in one (3.0%; Figure 1).

In 7 cases with desmoid tumor arising in the abdominal wall, 4 did not have mutations in exon 3 (wild type), 2 had codon 45 mutations (S45F and S45P) and one had a codon 41 mutation (T41A). In other sites, T41A was the most frequent mutation. The mean age of each mutation group was lower than that of the wild-type group, and the mean tumor size of each mutation group was larger than that of wild-type. However, there were no significant differences in gender (p = 0.67), age (p = 0.57), tumor size (p = 0.47), or site (p = 0.23) between the groups (Table 2).

**Table 2:** Relationship between CTNNB1 mutation status with clinical and pathological characteristics, and efficacy of meloxicam.

	Mutation status				
Variables	WT (n = 12)	T41A (n = 16)	S45F (n = 4)	S45P (n = 1)	P value
Gender (Female/Male)	7/5	12/4	2/2	1/0	0.67
Mean age, year	45.0	40.7	38.5	26.0	0.57
Mean Size, mm	74.7	82.4	106.5	118.0	0.47
Site					0.23
Abdominal wall	4	1	1	1	
Other trunk	2	5	2	0	
Neck	1	3	1	0	
Extremities	5	7	0	0	
Efficacy of meloxicam					0.053
Favorable group (CR, PR, SD)	8	11	0	1	
Unfavorable group (PD)	4	5	4	0	
β-catenin nuclear positivity^a^					0.035
Moderate (2+)	5	11	0	0	
Strong (3+)	7	5	4	1	

WT; wild type, CR; complete response, PR; partial response, SD; stable disease, PD; progressive disease.

a No cases showed negative (0) or weak (1+) positive.

b S41F vs other type.

**Figure 2:**
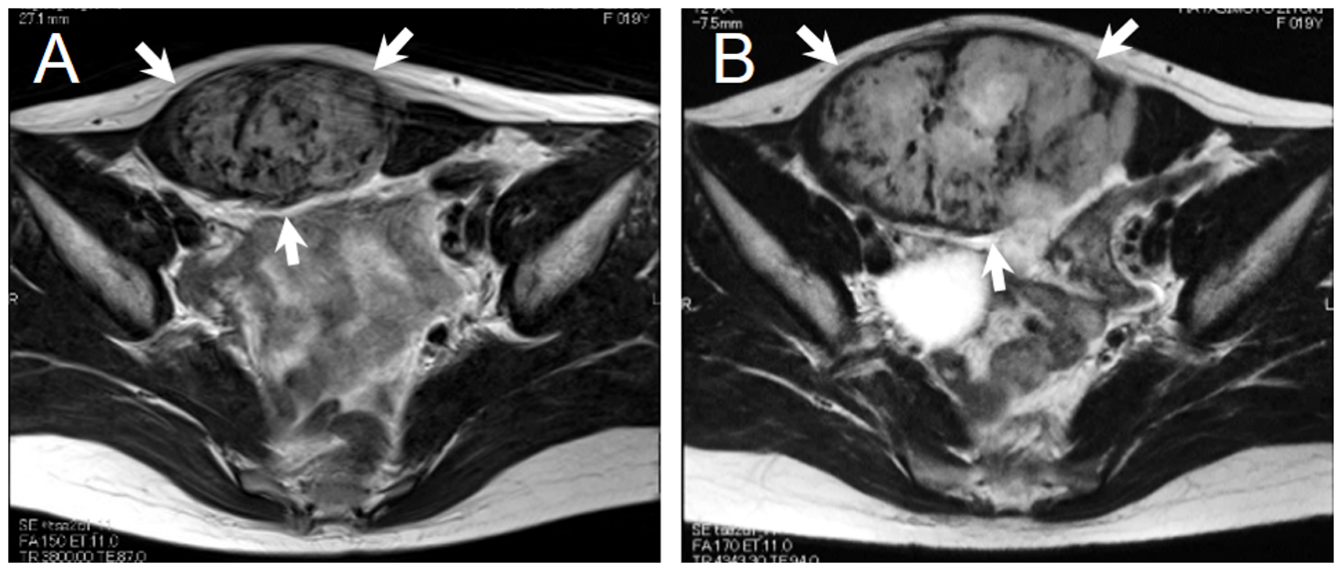
Twenty year-old woman with a desmoid tumor arising in the abdominal wall. Evaluation for efficacy of meloxicam according to RECIST criteria was PD. This tumor had S45F mutation. (A) A T2-weighed axial MRI of desmoid (arrows) at the first visit. (B) A T2-weighed axial MRI of desmoid (arrows) at the end of meloxicam treatment.

**Figure 3:**
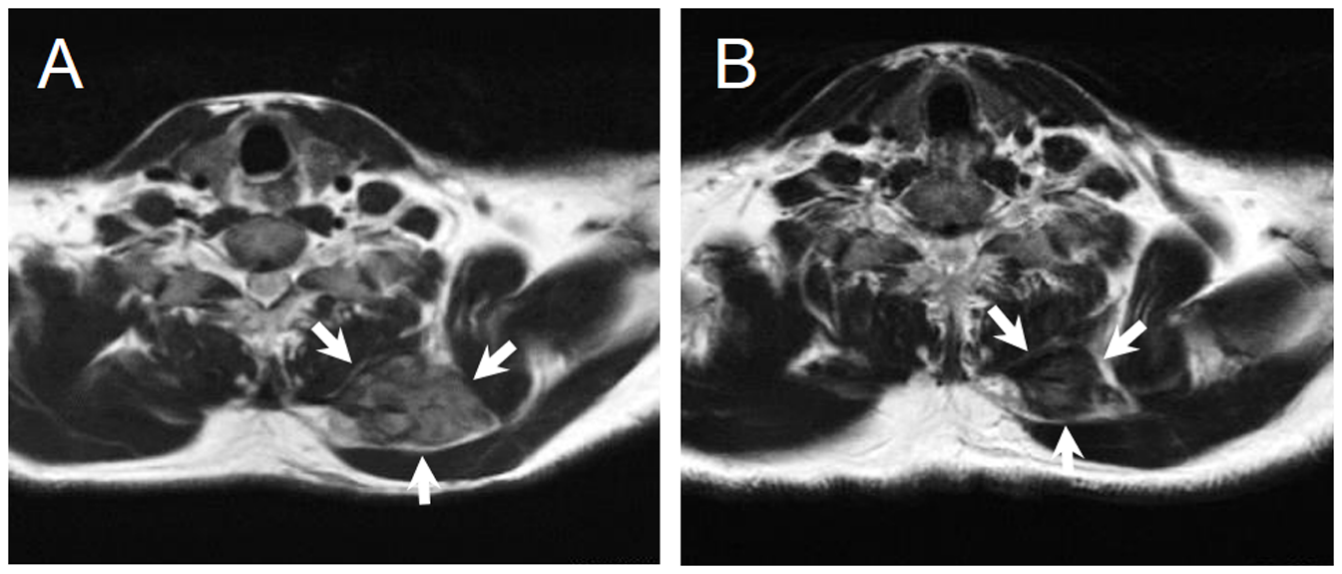
Forty-four year-old man with a desmoid tumor arising in the left posterior neck region. Evaluation for efficacy of meloxicam according to RECIST criteria was PR. This tumor had T41A mutation. (A) A T2-weighed axial MRI of desmoid (arrows) at the first visit. (B)A T2-weighed axial MRI of desmoid (arrows) at last follow-up.

In analyses of the correlation of mutation status of CTNNB1 and efficacy of meloxicam treatment, mutation status showed a trend to associate with efficacy (p = 0.053, Table 2). Focusing on the S45F mutation, all 4 cases with 45F mutation showed PD, and no cases in the favorable group (0/20 cases) had S45F mutation (p = 0.017, Table 3), whereas cases with T41A or wild type did not show a significant association with the efficacy of meloxicam. Representative cases are shown in Figures 2 and 3.

**Table 3:** S45F mutation status and efficacy of meloxicam.

	S45F (+)	S45F(−)
Favorable group (CR, PR, SD)	0	20
Unfavorable group (PD)	4	9

P = 0.017.

### Correlation of nuclear β-catenin staining and mutation type

As reported previously [23], positive nuclear staining for β-catenin was observed in all 33 cases, and there was no case with weak positive staining (1+). Strong positive (3+) indicated a poor prognosis in comparison with moderate (2+) staining (p = 0.032). There was a significant correlation between the nuclear positivity of β-catenin and CTNNB1 mutation status (p = 0.035), and all four cases with S45F mutation exhibited strong positive (3+) staining (Table 2).

## Discussion

Dysregulation of the Wnt/β-catenin signaling pathway is a common molecular event in desmoid tumors [25]. The nuclear accumulation of β-catenin caused by CTNNB1 or APC mutation subsequently activates T-cell factor, which in turn causes transcription of target genes such as c-MYC and cyclin D [25], [26]. In practice, mutations of CTNNB1 exon3 gene are found in a wide variety of human cancers, and occur between codon 32 and 45, the site of phosphorylation by GSK3β or CK1α [15], [27]. Regarding desmoid tumors, β-catenin mutations are restricted to exon 3 of CTNNB1, and several larger studies have described a high frequency of mutations (64%–85%) in codons 41 and 45 [16]–[19], [28]. In this study, we used not only frozen specimens but also paraffin-embedded tissue extracts, as described in other reports [29], [30]. CTNNB1 point mutations were observed in 21 of the 33 cases (64%). The majority (76%) of them were T41A mutations, whereas the others were codon 45 (S45F and S45P). These results are consistent with the results of previous reports that showed T41A to be the predominant type of mutation [17], [31]. Thus these two specific mutations (T41A and S45F) may play crucial roles in the pathogenesis of desmoid tumors.

Several reports have focused on the correlation between CTNNB1 mutation type and local recurrence after surgery in desmoid tumors [17], [19], [28]. Colombo et al. reported that mutation status was the only poor prognostic factor predictive of local recurrence of desmoid tumors in an analysis of 179 surgical cases [28]. Lazar et al. showed that the five-year recurrence-free survival was significantly worse in 45F-mutated desmoids than either 41A-mutated or nonmutated ones [17]. In contrast, two other reports did not identify any association between mutation type and recurrence risk after surgery [19], [32]. Mullen et al. showed that 2-year recurrence rates in mutated tumors (64%) were slightly worse than those in wild-type (77%), although the difference failed to reach statistical significance [32].

In our study, all four cases with S45F mutation exhibited PD, indicating that S45F status is a significant poor prognosticator for meloxicam treatment. In tumors with other mutation status including T41A mutation and wild-type, the efficacy of meloxicam could not be predicted. Although several previous studies reported the predictive value of CTNNB1 mutation type for the outcome of surgical treatment, the current study analyzed for the first time the significance of CTNNB1 mutations in the prediction of efficacy for conservative treatment. Recently, several reports proposed treatment algorithms for patients with desmoid tumors, mainly based on conservative therapy including a “wait and see” policy [10], [33], although molecular determinants were not described in these studies. As the current study indicated, CTNNB1 mutation type may be a possible determinant for successful conservative treatment including meloxicam treatment and “wait and see” policy.

The nuclear expression of β-catenin has increasingly been used in the differential diagnosis of spindle cell neoplasms because of the high positivity rate of desmoid tumors [13], [34], [35]. Results of these previous reports are consistent with those of our study, showing that all cases showed equal or higher than moderate (2+) positive staining of nuclear β-catenin expression. Several previous studies reported the significance of nuclear expression of β-catenin in surgically treated patients with desmoid tumors [17], [36]. However, the results of these studies are controversial, probably because immunohistochemical assessment may depend on the various parameters such as the antibody, technique and/or observation methods selected. The staining intensity may vary at different times, and be heterogeneous in different areas of the same tumors. Our previous study for the first time demonstrated that nuclear expression of β-catenin is a significant prognosticator for conservative treatment with meloxicam [23]. The current study showed a positive correlation between nuclear β-catenin staining and S45F mutation. In contrast, Lazar et al. reported that cases with CTNNB1 S45F mutations exhibited a less intense β-catenin staining compared to those with T41A mutations [17]. Taking these findings together, although immunohistochemical evaluation serves as a useful reference to predict the outcome of surgery or conservative treatment for patients with desmoid tumors, more definitive prognosticators, including mutation type as shown in the current study, are also required.

The limitations of this study include the fact that the sample size was relatively small compared to previous mutation studies with larger numbers of cases. However, the cohort of this study is composed of prospectively treated patients with meloxicam, while no previous studies have reported the significance of CTNNB1 mutation type in conservative treatment. The results derived from this study provide meaningful information. Second, CTNNB1 mutation was analyzed only in exon 3. Other significant mutations may exist in different areas of CTNNB1. Third, due to the small number of cases, we could not analyze the relationship between mutation status and period up to onset of efficacy for meloxicam treatment. Future studies with more accumulated cases with molecular analysis will be needed to clarify the further correlation between the prognosis and mutation status.

In conclusion, we demonstrate for the first time that S45F mutation of CTNNB1 may serve as a prognostic marker in patients with sporadic desmoid tumors treated with conservative treatment with meloxicam. Given the current shift in the treatment modality for patients with desmoid to conservative treatment, the identification of prognosticators for conservative treatment is more important than ever. Mutation status of CTNNB1 may be a promising tool to predict the efficacy of conservative treatment including meloxicam.
